# Mycoplasma pneumoniae-Associated Reactive Infectious Mucocutaneous Eruption Mimicking Atypical Stevens-Johnson Syndrome: A Case Report

**DOI:** 10.7759/cureus.112262

**Published:** 2026-07-08

**Authors:** Alexandru C Burbea, Pankaj Verlekar

**Affiliations:** 1 Acute Medicine, Hampshire Hospitals NHS Foundation Trust, Winchester, GBR; 2 Diabetes and Endocrinology, Hampshire Hospitals NHS Foundation Trust, Winchester, GBR

**Keywords:** mycoplasma pneumonia-associated mucositis, mycoplasma pneumoniae infection, ocular involvement, reactive infectious mucocutaneous eruption (rime), stevens-johnson syndrome (sjs)

## Abstract

Reactive infectious mucocutaneous eruption (RIME) is an infection-associated mucositis syndrome previously described under terms including incomplete Stevens-Johnson syndrome (SJS), Fuchs syndrome, and *Mycoplasma pneumoniae*-induced rash and mucositis (MIRM). It is characterised by an infectious prodrome, prominent mucosal involvement, and sparse or absent cutaneous disease. Severe ocular involvement may clinically mimic atypical Stevens-Johnson syndrome.

We report the case of a previously healthy woman in her 20s who developed coryzal symptoms and self-treated with ibuprofen, paracetamol, and pseudoephedrine before presenting with worsening cough, pyrexia, odynophagia, and bilateral conjunctival discharge. She received doxycycline and topical chloramphenicol but re-presented overnight with rapidly progressive lip swelling, dysphagia, oral mucosal ulceration, and severe bilateral ocular inflammation without cutaneous lesions. Recent exposure to non-steroidal anti-inflammatory medication and doxycycline created initial diagnostic uncertainty, while the preceding respiratory prodrome and skin-sparing mucositis supported infection-associated RIME.

Ophthalmological assessment demonstrated severe ocular surface inflammation with pseudomembrane formation, early symblepharon, and progressive corneal involvement, requiring tertiary transfer and amniotic membrane grafting. Initial chest radiography and respiratory multiplex polymerase chain reaction (PCR) testing were negative, including for *Mycoplasma pneumoniae*; however, subsequent serology returned positive for *Mycoplasma pneumoniae* IgM. Later drug challenge testing demonstrated tolerance to suspected medications, supporting an infection-associated rather than drug-induced aetiology.

This case highlights that *Mycoplasma pneumoniae*-associated RIME may mimic atypical Stevens-Johnson syndrome, particularly when severe ocular and oral mucositis occurs after recent medication exposure. Early dermatology and ophthalmology involvement, combined molecular and serological testing, and prompt ocular treatment are essential to reduce long-term ocular morbidity.

## Introduction

Stevens-Johnson syndrome (SJS) is an uncommon but serious mucocutaneous disorder characterised by epithelial necrosis, multisite mucositis, and variable epidermal detachment. It is most commonly drug-induced, although infection-associated mucosal-predominant presentations, particularly those linked to *Mycoplasma pneumoniae*, have long been described under overlapping terms including atypical SJS, incomplete SJS, Fuchs syndrome, and SJS without skin lesions [1-4].

More recently, reactive infectious mucocutaneous eruption (RIME) has been proposed as a broader term for infection-triggered mucositis with sparse or absent cutaneous involvement [5]. This terminology reflects the evolving understanding that some cases previously classified within the SJS/toxic epidermal necrolysis (TEN) spectrum are better distinguished by an infectious trigger, prominent mucosal disease, minimal skin involvement, and a generally more favourable prognosis [5,6]. *Mycoplasma pneumoniae*-induced rash and mucositis (MIRM) was previously described as distinct from SJS and erythema multiforme, and is now generally considered within the wider RIME spectrum [5,6].

Distinguishing RIME from atypical SJS can be challenging, particularly when severe ocular and oral mucositis occurs after recent medication exposure. Ocular involvement is clinically important because severe ocular surface inflammation may progress to pseudomembrane formation, symblepharon, corneal epithelial disease, and long-term visual morbidity. Early ophthalmology involvement and, in severe cases, amniotic membrane transplantation may reduce chronic ocular sequelae [7-9].

We present a case of serology-supported *Mycoplasma pneumoniae*-associated RIME with severe ocular and oral mucosal involvement, no cutaneous lesions, and recent exposure to non-steroidal anti-inflammatory medication and doxycycline, initially mimicking atypical SJS.

## Case presentation

A previously healthy woman in her 20s developed coryzal symptoms for approximately one week and self-treated with ibuprofen, paracetamol, and pseudoephedrine. Her medical history included childhood epilepsy, seizure-free for eight years, previous strabismus surgery, and a documented penicillin allergy. No recent travel was reported.

She initially attended the emergency department with worsening cough, odynophagia, pyrexia (39.3°C), and bilateral conjunctival discharge. She received an initial dose of doxycycline and was discharged with a course to complete, along with topical chloramphenicol eye drops.

**Figure 1:**
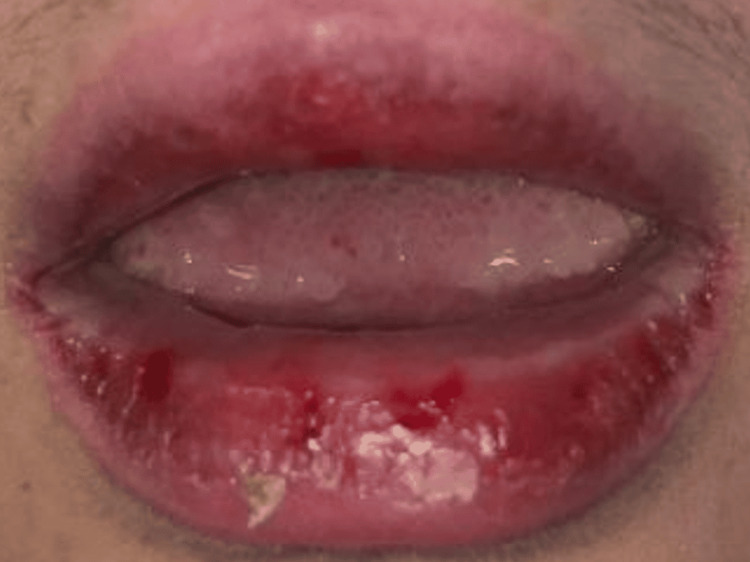
Marked lip swelling and mucosal erosions

**Figure 2:**
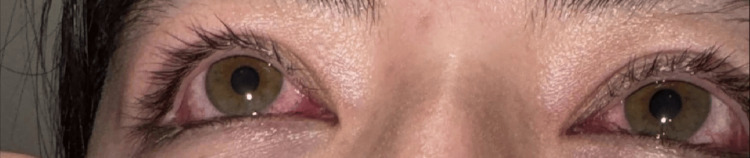
Bilateral conjunctival injection with periocular oedema

**Figure 3:**
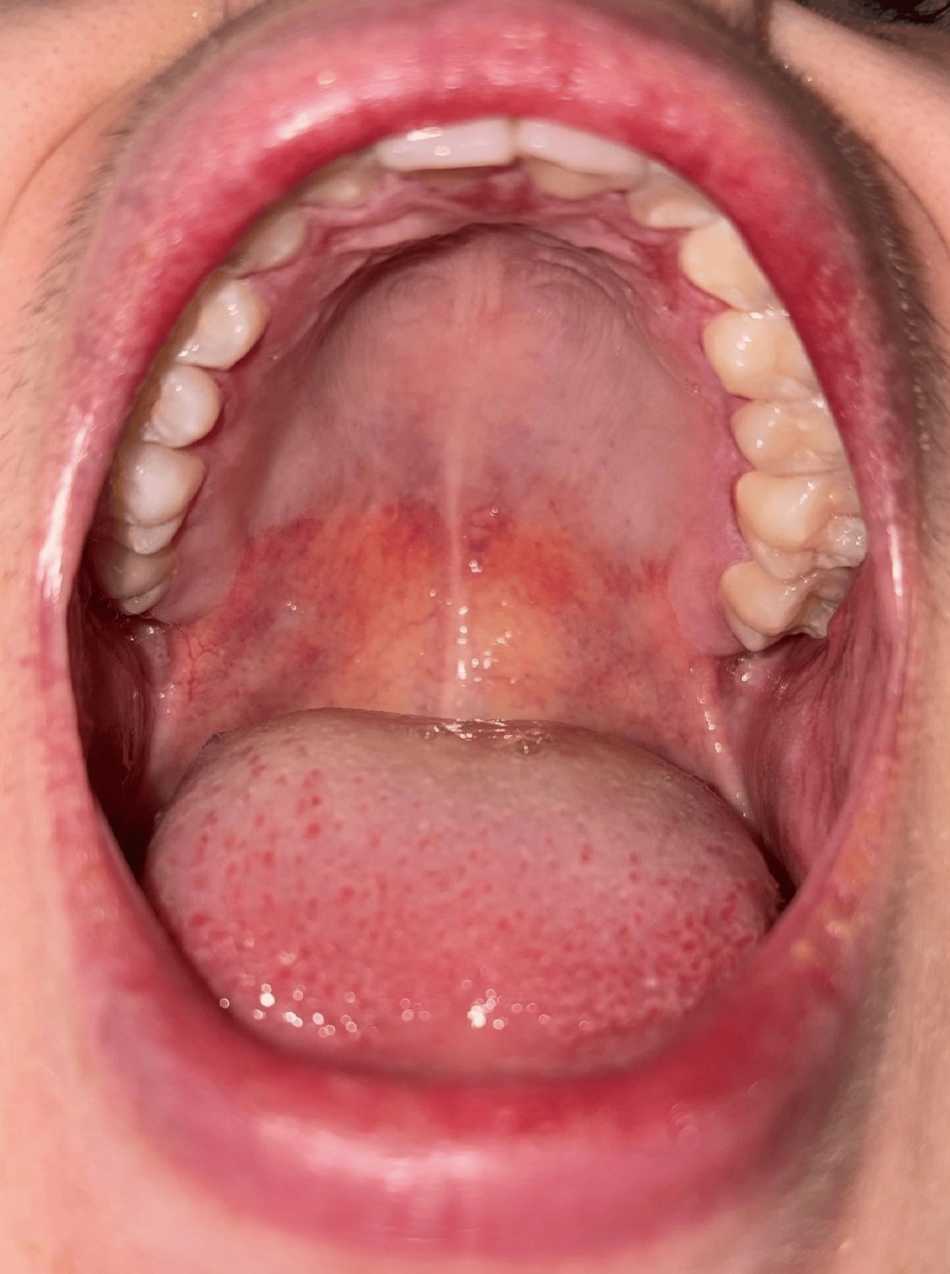
Diffuse palatal erythema with oral mucosal inflammation

**Figure 4:**
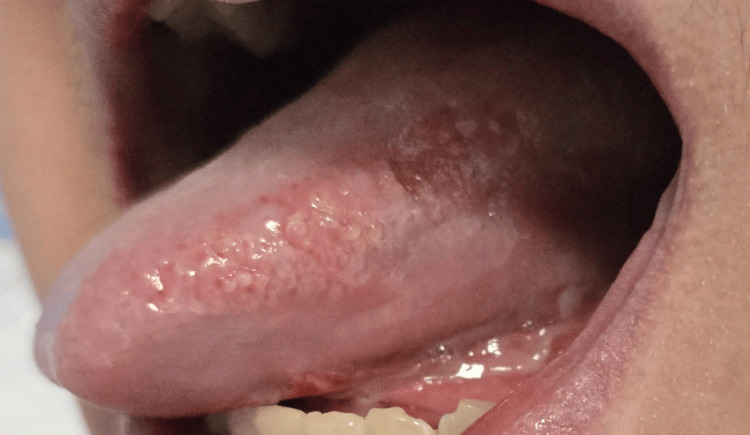
Oral mucosal lesions involving the tongue

She re-presented eight hours later with rapidly progressive lip swelling and severe mucosal involvement of the lips (Figure 1), accompanied by dysphagia and bilateral conjunctival inflammation (Figure 2). She was afebrile but fatigued, and examination revealed an erythematous pharynx (Figure 3), oral mucosal ulceration involving the tongue and lower labial mucosa (Figure 4), and cervical lymphadenopathy without cutaneous lesions. Otoscopic and nasal examinations were unremarkable aside from minor anterior epistaxis. She was admitted to the medical ward for observation and further investigations.

A summary of the patient's laboratory and microbiological investigations is presented in Table 1.

**Table 1:** Summary of investigations during hospital admission CRP: C-reactive protein, WCC: white cell count, PCR: polymerase chain reaction, EBV: Epstein-Barr virus, CMV: cytomegalovirus, HSV: herpes simplex virus, VZV: varicella-zoster virus, IgM: immunoglobulin M BioFire: multiplex respiratory pathogen PCR assay performed on nose and throat samples Laboratory values are reported using standard SI units

Test	Result	Laboratory reference range
CRP	117 mg/L	0-4.9 mg/L
WCC	10.2 × 10⁹/L	4.0-10.0 × 10⁹/L
Procalcitonin	0.12 ng/mL	0-0.25 ng/mL
Chest X-ray	Normal	-
Viral and atypical infection screen	EBV, CMV, leptospira, measles PCR, VZV DNA, HSV 1/2 PCR all negative	-
Sexually transmitted infection screen	*Chlamydia* and *Neisseria gonorrhoeae* PCR negative	-
Multiplex respiratory pathogen PCR (BioFire)	Negative (including *Mycoplasma*)	-
Blood cultures	No growth	-
Urine cultures	No growth	-

Empirical treatment for suspected lower respiratory tract infection was initiated with intravenous co-trimoxazole due to a documented penicillin allergy, alongside topical chloramphenicol eye drops. Following microbiology advice, clarithromycin 500 mg orally every 12 hours was subsequently added.

Ophthalmology review documented that the patient's red eyes had started eight to nine days earlier, before antibiotic exposure, and that her blurred vision had progressively worsened. Initial ocular assessment demonstrated severe bilateral conjunctival hyperaemia, subconjunctival haemorrhage, and central corneal epithelial sloughing in both eyes. Visual acuity deteriorated to count-fingers bilaterally. There were no corneal infiltrates, the anterior chambers were deep and quiet, and there was no relative afferent pupillary defect. The clinical impression was severe ocular surface disease, with concern for SJS spectrum ocular involvement in view of the typical lip appearance and ocular findings.

Preservative-free lubricating eye drops and chloramphenicol ointment were commenced, and dermatology review was recommended. On ophthalmological reassessment the following day, the ocular disease had progressed, with pseudomembrane formation involving the upper and lower eyelids bilaterally, loose corneal epithelium in both eyes, and early nasal symblepharon formation. The pseudomembranes were removed from the upper and lower eyelids bilaterally. A tertiary corneal specialist opinion was sought because of the rapid progression and risk of permanent ocular surface damage.

Topical therapy was intensified to include hourly dexamethasone 0.1% eye drops, hourly levofloxacin eye drops, preservative-free chloramphenicol drops, intensive lubricating drops, and Xailin Night ointment. Following pseudomembrane removal and aggressive topical therapy, visual acuity improved in the left eye to 0.4, and no lid adhesions were visible on repeat review. However, given the preceding count-fingers visual acuity, bilateral corneal epithelial sloughing, pseudomembrane formation, and early symblepharon, she remained at high risk of progressive ocular surface scarring.

Given the rapid progression of ocular surface disease, she was subsequently transferred to a tertiary ophthalmology centre, where she underwent ocular amniotic membrane grafting and was commenced on systemic corticosteroids, topical ophthalmic corticosteroids, and intravenous immunoglobulin.

Slit-lamp photographic documentation of the acute ocular findings, including pseudomembranes and early symblepharon, was not obtained and is therefore unavailable. Formal intraocular pressure measurements, fluorescein staining grades, and objective ocular severity scores at initial presentation were not consistently available from the contemporaneous clinical records; therefore, ocular severity is described using the documented specialist ophthalmology findings and subsequent clinical course.

She remained under multidisciplinary inpatient management with close ophthalmological monitoring and supportive care and was discharged after a total hospital stay of 18 days. A summary of the medications prescribed on discharge from the tertiary ophthalmology centre is provided in Table 2.

**Table 2:** Medications on discharge from the tertiary centre Medications prescribed on discharge following tertiary ophthalmology admission. Dose, route, frequency, and duration are shown as documented on the discharge medication chart. All ophthalmic treatments were administered to both eyes unless otherwise stated.

Category	Medication	Dose, route, and duration
Ophthalmic	Dexamethasone 0.1% eye drops	One drop to both eyes hourly for 7 days
	Levofloxacin eye drops	One drop to both eyes four times daily for 7 days
	Sodium hyaluronate 0.2% eye drops	One drop to both eyes hourly for 28 days
	Thealoz Duo (trehalose 3%/sodium hyaluronate 0.15%) eye drops	One drop to both eyes hourly for 28 days
	Xailin Night eye ointment	Applied to both eyes nightly for 28 days
Oral/mucosal	Chlorhexidine gluconate 0.2% mouthwash	10 mL oral rinse twice daily for 7 days
	Betamethasone sodium phosphate mouthwash	500 micrograms dissolved in water and used once daily as a mouthwash (not swallowed)
	Nystatin oral suspension	1 mL four times daily for 10 days
Systemic/other	Oral prednisolone	30 mg daily for 7 days, then 20 mg daily for 7 days, then 10 mg daily for 7 days, then stopped
	Oral lansoprazole	15 mg once daily for 28 days
	Oral fexofenadine hydrochloride	180 mg once daily for 28 days
	Topical Epaderm ointment	Applied to skin once daily for 28 days

Serological investigations sent earlier during admission later returned positive for *Mycoplasma pneumoniae* IgM, supporting *Mycoplasma pneumoniae*-associated RIME as the most likely diagnosis, rather than a drug-induced SJS spectrum reaction.

Outcome and follow-up

Immunological evaluation, including dermatology-supervised drug challenge protocols, demonstrated tolerance to previously suspected medications, including non-steroidal anti-inflammatory drugs and antibiotics. This made a drug-induced SJS spectrum reaction unlikely and supported *Mycoplasma pneumoniae* infection as the most likely precipitating factor for the patient's RIME-like presentation.

The patient remained under regular ophthalmology follow-up. At review one week after discharge, corneal inflammation persisted but showed signs of improvement. A superotemporal symblepharon was noted in the right eye. On the same day, the corneal rings, bandage contact lens, and corneal sutures were removed, and plans were made for continued monitoring of corneal healing and ocular surface stability.

One month later, the patient re-presented with a progressively enlarging right eyelid mass. Ophthalmology assessment documented right symblepharon to the lid, a right eyelid cyst, and bilateral chronic cicatrising conjunctivitis. She underwent right incision and drainage of the cyst under general anaesthesia and continued regular ophthalmology follow-up.

At review five months post-discharge, the patient developed ocular hypertension attributed to topical corticosteroid therapy. This resolved following cessation of the steroid drops, and visual acuity remained preserved.

Nine months after discharge, intraocular pressures remained within the normal range, and visual acuity was stable. The patient continues to attend ophthalmology outpatient follow-up every two to three months and remains on topical lubricants, anti-inflammatory agents, and tear substitutes to support long-term ocular surface stability.

## Discussion

This case illustrates *Mycoplasma pneumoniae*-associated reactive infectious mucocutaneous eruption (RIME) with severe ocular and oral mucosal involvement, initially mimicking atypical Stevens-Johnson syndrome (SJS). The absence of cutaneous lesions, preceding respiratory prodrome, later positive *Mycoplasma pneumoniae* IgM serology, and subsequent tolerance of suspected medications support an infection-associated RIME phenotype rather than a drug-induced SJS spectrum reaction. However, the severity of ocular disease, marked lip involvement, recent medication exposure, and early concern for SJS spectrum ocular surface disease explain why atypical SJS was considered during the acute presentation.

The terminology surrounding infection-associated mucosal-predominant disease has evolved. Cases with severe mucositis and minimal or absent skin involvement were previously described as incomplete SJS, Fuchs syndrome, SJS without skin lesions, or *Mycoplasma pneumoniae*-associated atypical SJS [1-4]. More recently, *Mycoplasma pneumoniae*-induced rash and mucositis (MIRM) was described as distinct from SJS and erythema multiforme, and the broader term RIME has been proposed to include similar mucocutaneous eruptions triggered by *Mycoplasma pneumoniae* and other infections [5,6]. In this context, our patient's presentation is best understood as *Mycoplasma pneumoniae*-associated RIME mimicking atypical SJS, rather than classical drug-induced SJS.

Distinguishing RIME from atypical SJS can be difficult in the acute setting, particularly when recent medication exposure complicates the clinical picture. In this case, the patient self-treated with non-steroidal anti-inflammatory drugs and later received doxycycline before rapid mucosal deterioration, making both drug-related SJS spectrum disease and infection-associated mucositis plausible early considerations. The chronology, however, favoured infection-associated disease: respiratory and ocular symptoms preceded antibiotic exposure, the illness followed an approximately one-week respiratory prodrome, no cutaneous lesions developed, *Mycoplasma pneumoniae* IgM later returned positive, and drug challenge testing subsequently demonstrated tolerance of suspected medications. These features support RIME while explaining why an SJS spectrum reaction remained an important early differential diagnosis.

Diagnosis of *Mycoplasma pneumoniae*-associated RIME may be complicated by limitations in testing. There is no single confirmatory laboratory test for RIME, and investigations are usually directed toward identifying an infectious trigger [5]. In this patient, chest radiography was normal, and multiplex respiratory pathogen PCR was negative, including for *Mycoplasma pneumoniae*, but subsequent serology returned positive for *Mycoplasma pneumoniae* IgM. This supports the need to combine clinical pattern recognition with both molecular and serological testing when infection-associated mucositis is suspected, particularly when respiratory PCR is negative, but the clinical phenotype remains suggestive.

Ocular involvement was the major determinant of morbidity in this case. The patient developed severe bilateral conjunctival hyperaemia, subconjunctival haemorrhage, central corneal epithelial sloughing, count-fingers visual acuity bilaterally, pseudomembranes involving the upper and lower eyelids, loose corneal epithelium, and early nasal symblepharon formation. Severe ocular surface inflammation in SJS/TEN spectrum disease and related mucocutaneous eruptions may lead to conjunctival scarring, symblepharon, corneal epithelial defects, and long-term visual impairment. Early ophthalmological assessment, intensive lubrication, topical corticosteroids, antimicrobial cover, pseudomembrane removal, and close monitoring are therefore essential. In severe or progressive ocular surface disease, amniotic membrane transplantation may reduce chronic ocular sequelae by suppressing inflammation, promoting epithelial healing, and limiting conjunctival fibrosis [7-9].

Management of RIME remains incompletely standardised. Supportive care, hydration, analgesia, mucosal care, and treatment of any identified infectious trigger remain central [5]. Systemic corticosteroids and intravenous immunoglobulin are reported in severe cases, particularly where there is extensive mucosal or ocular involvement, but evidence remains limited, and treatment decisions are often extrapolated from SJS/TEN experience [5]. In this case, escalation to systemic corticosteroids, intravenous immunoglobulin, intensive topical ophthalmic therapy, and amniotic membrane grafting occurred in the context of severe and rapidly progressive ocular surface disease. Although RIME generally has a more favourable prognosis than classical drug-induced SJS/TEN, significant ocular sequelae may still occur, as demonstrated by this patient's prolonged ophthalmology follow-up, symblepharon, chronic cicatrising conjunctivitis, conjunctival cyst requiring drainage, and steroid-related ocular hypertension.

This case reinforces several clinical lessons. First, RIME should be considered in patients with severe oral and ocular mucositis, minimal or absent cutaneous involvement, and a preceding respiratory prodrome, even when recent medication exposure creates concern for atypical SJS. Second, negative respiratory PCR testing does not exclude *Mycoplasma pneumoniae*-associated disease, and serology may provide important supportive evidence. Finally, early multidisciplinary involvement, particularly dermatology and ophthalmology input, is critical because severe ocular RIME may progress rapidly and require specialist ocular surface-directed treatment to reduce long-term morbidity.

## Conclusions

This case illustrates *Mycoplasma pneumoniae*-associated reactive infectious mucocutaneous eruption (RIME) with severe ocular and oral mucosal involvement in the absence of cutaneous lesions, initially mimicking atypical Stevens-Johnson syndrome. The diagnosis was challenging because recent exposure to non-steroidal anti-inflammatory medication and doxycycline created early concern for a drug-related SJS spectrum reaction, while the preceding respiratory prodrome, positive *Mycoplasma pneumoniae* serology, absence of skin involvement, and later tolerance of suspected medications supported an infection-associated aetiology. Clinicians should consider RIME in patients presenting with multisite mucositis after respiratory symptoms, even when respiratory PCR testing is negative or recent medication exposure complicates the initial assessment. Early multidisciplinary involvement, particularly dermatology and ophthalmology input, is essential to minimise the risk of long-term ocular morbidity.
